# A Bibliometric Analysis of Exosomes in Cardiovascular Diseases From 2001 to 2021

**DOI:** 10.3389/fcvm.2021.734514

**Published:** 2021-08-25

**Authors:** Dan Ma, Baoyi Guan, Luxia Song, Qiyu Liu, Yixuan Fan, Lin Zhao, Tongxin Wang, Zihao Zhang, Zhuye Gao, Siming Li, Hao Xu

**Affiliations:** ^1^Xiyuan Hospital, China Academy of Chinese Medical Sciences, Beijing, China; ^2^Graduate School, Beijing University of Chinese Medicine, Beijing, China; ^3^National Clinical Research Center for Chinese Medicine Cardiology, Xiyuan Hospital, China Academy of Chinese Medical Sciences, Beijing, China

**Keywords:** cardiovascular disease, CiteSpace, VOSviewer, bibliometrics, cardiovascular diseases

## Abstract

**Background:** Exosomes in cardiovascular diseases (CVDs) have become an active research field with substantial value and potential. Nevertheless, there are few bibliometric studies in this field. We aimed to visualize the research hotspots and trends of exosomes in CVDs using a bibliometric analysis to help understand the future development of basic and clinical research.

**Methods:** The articles and reviews regarding exosomes in the CVDs were culled from the Web of Science Core Collection, and knowledge maps were generated using CiteSpace and VOSviewer software.

**Results:** A total of 1,039 articles were included. The number of exosome articles in the CVDs increased yearly. These publications came from 60 countries/regions, led by the US and China. The primary research institutions were Shanghai Jiao Tong University and Nanjing Medical University. *Circulation Research* was the journal and co-cited journal with the most studies. We identified 473 authors among which Lucio Barile had the most significant number of articles and Thery C was co-cited most often. After analysis, the most common keywords are myocardium infarction, microRNA and mesenchymal stem cells. Ischemic heart disease, pathogenesis, regeneration, stem cells, targeted therapy, biomarkers, cardiac protection, and others are current and developing areas of study.

**Conclusion:** We identified the research hotspots and trends of exosomes in CVDs using bibliometric and visual methods. Research on exosomes is flourishing in the cardiovascular medicine. Regenerative medicine, exosome engineering, delivery vehicles, and biomarkers will likely become the focus of future research.

## Introduction

Exosomes are a subset of nanosized extracellular vesicles of 40–160 nm in diameter. They possess the same lipid bilayer structure as the origin cells and are rich in bioactive substances, including DNA, RNA, lipid, proteins, metabolites, and other molecules. Nearly all types of living cells release exosomes (1–3). Exosomes were once considered vehicles or “garbage bags,” responsible for removing cell debris, including redundant intracellular organelles, for retaining cellular homeostasis (4). In the mid-1990's, exosomes secreted by immune cells were thought to be related to immune regulation. Over time, exosomes widely present in various body fluids came to be viewed as functional membrane vesicles that mediate intercellular communication by transferring bioactive materials in normal physiology and various diseases and acting as signaling molecules in homeostatic processes or as a result of pathological progression (5–10).

Several studies showed that exosomes participate in cardiovascular diseases (CVDs) (11–14). Several animal experiments demonstrated that exosomes carrying specific functional substances inhibit cardiomyocyte apoptosis and promoting angiogenesis to improve ventricular function and reduce the area of myocardial infarction (MI) (15, 16). Fu et al. found that exosomes rich in miR-338 derived from MSCs reduced cardiomyocyte apoptosis and improved cardiac function in rats with MI by regulating the MAP3K2/JNK signaling pathway (17). Another study showed that HIF-1α overexpression in exosomes mediated cardioprotection in MI by enhancing angiogenesis (18).

Various contents of exosomes represent their cell sources and reflect the physiological and pathological conditions of the origin cells; these cargos can be used as non-invasive diagnostic biomarkers. After MI, the components of circulating exosomes change significantly. A study showed that miRNAs are released when cardiomyocytes are injured, and circulating miR-133a derived from the MI area and the marginal region elevate at an early stage after MI, which can be detected earlier than creatine phosphokinase and cardiac troponin T (19).

As regenerative medicine becomes clinically feasible, there has been extensive interest in stem cell-based therapies for CVDs. However, numerous side effects and low survival of implanted stem cells limited their therapeutic potency (20–26). Recently, evidence suggested that stem cells exert therapeutic effects in a paracrine manner through exosomes (27). Exosomes protect substances from degradation and transport material to recipient cells without causing toxicity or adverse immune responses (28).

Bibliometric analysis focuses on the literature systems and characteristics and has been widely used to understand the knowledge structure and explore developmental trends using qualitative and quantitative analysis of the scientific literature (29, 30). The bibliometric analysis method allows quantitative measurement of the domain outline distribution and the relationship and clustering of a study. In addition to describing and predicting the future development of a particular research area, the contributions of various authors, institutions, countries, and journals can be compared. This analytical approach plays a vital role in developing guidelines, understanding research hotspots, and evaluating research trends (31). Digestive system diseases (32), cancer (33), rheumatic system diseases (34), and nervous system diseases (35), have been studied using this method.

The present study explores the hotspots and developmental trends of exosome research in cardiovascular science over the past 20 years and draws maps of scientific knowledge using CiteSpace and VOSviewer software. The aim was to provide a basis for scientific research into CVDs.

## Methods

### Data Collection

**Figure 1:**
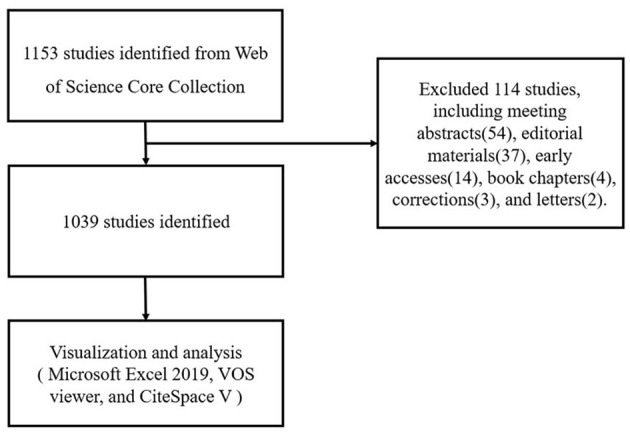
Flowchart of literature selection.

Literature was extracted from the Science Citation Index Expanded and Social Science Citation Index of Web of Science Core Collection and was downloaded within 1 day on June 5, 2021. The search terms were as follows: TS = (“cardiovascular” OR “heart”) AND TS = (exosomes), and the dates of the search were June 5, 2001, to June 5, 2021, resulting in 1,153 records. We eliminated invalid documents, including meeting abstracts (54), editorial materials (37), early accesses (14), book chapters (4), corrections (3), and letters (2). A total of 1,039 records retrieved were divided into eight document types, of which articles (639) accounted for 55.42% of the total, followed by reviews (400, 34.70%). The retrieved papers were exported and saved as plain text files, and stored in download_txt format (Figure 1).

### Data Analysis

All valid documents retrieved from Web of Science Core Collection were converted to Microsoft Excel 2019, VOSviewer, and CiteSpace to perform visual analysis.

VOSviewer is a scientometrics network analysis software developed by the Center for Science and Technology Research at Leiden University in the Netherlands. It provides visual analysis and creates maps based on network data. It can construct network diagrams of academic publications, scientific journals, authors, research institutions, countries, and keywords. The items in these networks can be connected by co-citation links, co-occurrence, citation, and bibliographic coupling. VOSviewer software provides three visualization maps: network, overlay and density visualizations (36). The core idea of the software design is co-occurrence clustering, which indicates that they are related. There are several correlations with varying intensities and directions. Based on the measurement index clustering of relationship intensity and direction, various groups can be found. Although VOSviewer is primarily used for bibliometrics, it may also create virtually any type of map of web data. Its most prominent feature is displaying graphics and is suitable for large-scale data (37).

CiteSpace software is a citation visualization analysis software developed by Professor Chen Chaomei of Drexel University using Java language based on scientometrics and data visualization (38). It presents the structure, laws, and distribution of scientific knowledge using data mining, information analysis, and atlas drawing. Knowledge mapping is a new sub-field of information technology. It is used to visualize research hotspots and evolution processes intuitively and forecast the developmental trends of each field. It is an effective method to analyze big data (37, 39).

We used Microsoft Office Excel 2019 to analyze the articles. We used CiteSpace and VOSviewer software to analyze the distribution of countries/regions visually, authors and co-cited authors, journals and co-cited journals, co-cited references, keyword cluster analysis, and timelines.

## Results

### The Trend of Publication Outputs

**Figure 2:**
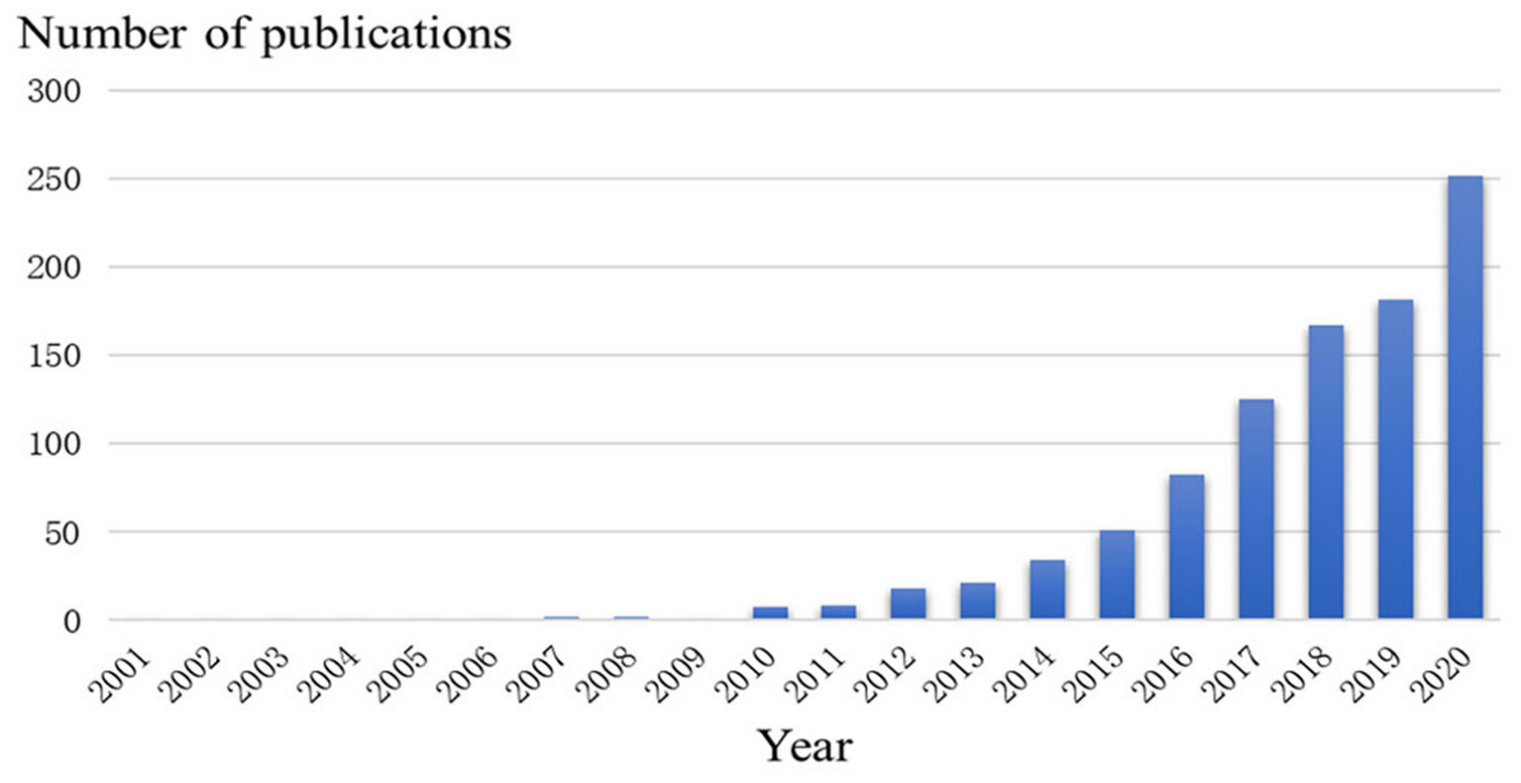
Trends of exosomes published in cardiovascular studies over the past 20 years.

The number of publications in a specific period reflects the developmental trends of research in a field (Figure 2). From 2001 to 2020, the number of studies published on exosomes in cardiovascular research showed an overall upward trend. From 2007 to 2009, the number of articles was relatively low, and the research and development of exosomes in cardiovascular science were in an embryonic stage. From 2010 to 2016, the annual growth rate of the number of published papers increased steadily. From 2017 to 2020, the number of articles published on exosomes in cardiovascular medicine increased significantly, and the total number of outputs in 2020 reached 251. It can be seen that a growing number of scholars have begun to pay much attention to the potential of exosomes in cardiovascular fields.

### Distribution of Countries/Regions and Institutions

**Figure 3:**
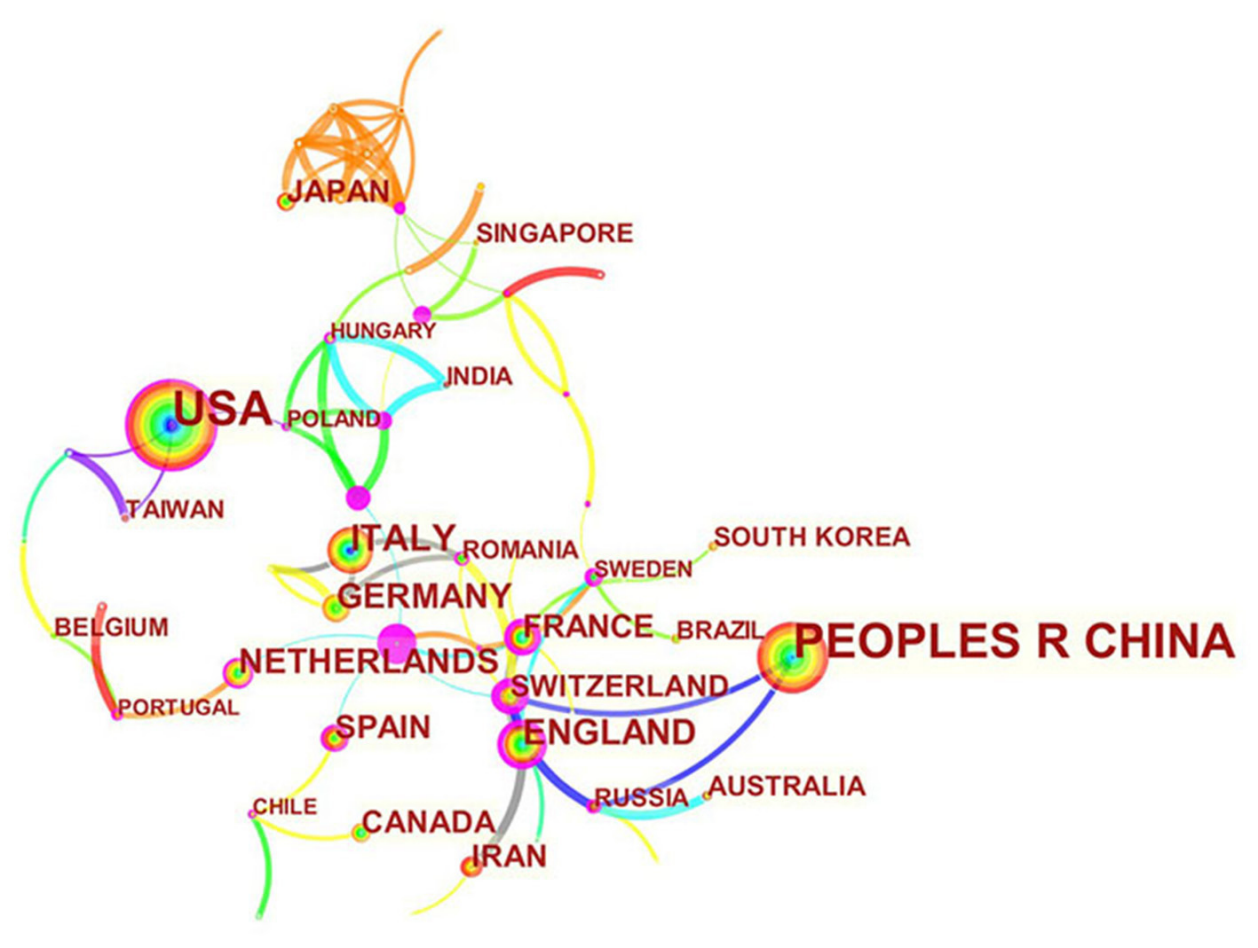
Distribution of publications from different countries.

**Figure 4:**
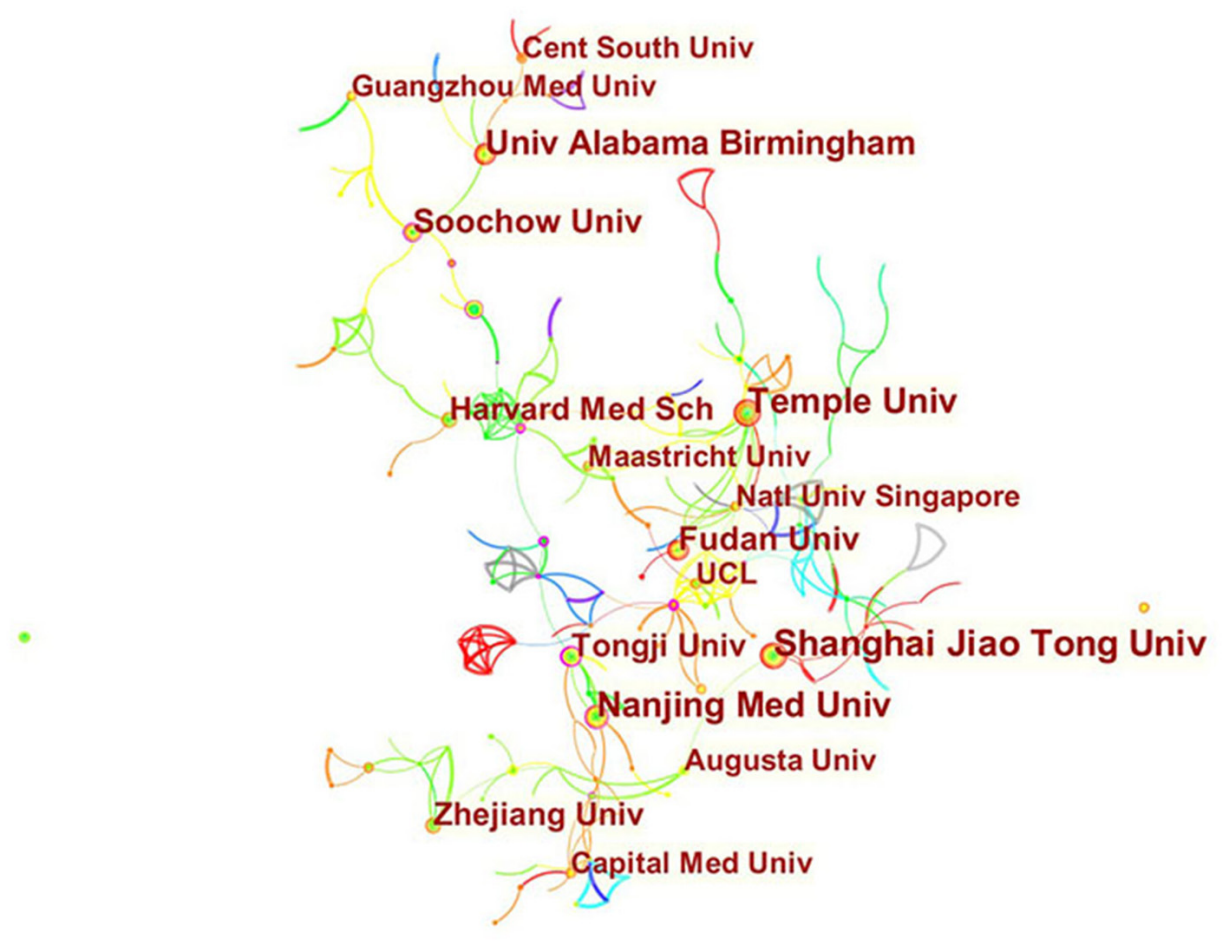
Distribution of publications from different institutions.

A total of 1,039 articles were published by 351 institutions in 60 countries/regions. As shown in Table 1, the most significant number of publications came from the US (343, 33.01%) and China (333, 32.05%), followed by Italy (77, 7.41%), England (62, 5.97%), and Germany (51, 4.91%). The total number of articles from these two highest-ranked countries was more than half of the total. Several countries and institutions, such as France (0.38), England (0.21), Netherlands (0.19), Tongji Univ (0.23), and Nanjing Med Univ (0.19), showed high centrality, circled in purple in Figures 3, 4. This finding suggests that the study of exosomes in these countries and institutions may have played a critical role in cardiovascular research. Each node represents a country, and the size of the node is proportional to the number of articles published. The lines between nodes represent cooperation between countries; denser lines correspond to closer cooperation. Figure 3 shows 60 nodes and 85 connections with a network density of 0.048, and Figure 4 shows 351 nodes and 396 connections with a network density of 0.0064. There is active cooperation among countries and institutions, including China, Russia and Switzerland, Nanjing Med Univ., Tongji Univ., and Capital Med Univ.

**Table 1:** Distribution of publications from different countries and institutions.

**No**.	**Country**	**Year**	**Centrality**	**Count (%)**	**Institution**	**Year**	**Centrality**	**Count (%)**
1	US	2009	0.14	343 (33.01%)	Shanghai Jiao Tong Univ (China)	2017	0.06	21 (2.02%)
2	Peoples R China	2012	0.00	333 (32.05%)	Nanjing Med Univ (China)	2017	0.19	20 (1.92%)
3	Italy	2010	0.03	77 (7.41%)	Temple Univ (US)	2016	0.06	20 (1.92%)
4	England	2008	0.21	62 (5.97%)	Univ Alabama Birmingham (US)	2018	0.06	17 (1.64%)
5	Germany	2007	0.03	51 (4.91%)	Soochow Univ (China)	2015	0.10	15 (1.44%)
6	Netherlands	2007	0.19	51 (4.91%)	Fudan Univ (China)	2018	0.01	15 (1.44%)
7	Spain	2008	0.18	47 (4.52%)	Harvard Med Sch (US)	2017	0.05	14 (1.35%)
8	France	2003	0.38	42 (3.90%)	Zhejiang Univ (China)	2017	0.04	13 (1.25%)
9	Canada	2013	0.00	36 (3.46%)	Tongji Univ (China)	2017	0.23	13 (1.25%)
10	Iran	2011	0.06	34 (3.27%)	Natl Univ Singapore (Singapore)	2017	0.01	12 (1.15%)

### Journals and Co-cited Academic Journals

We found that 1,039 articles related to exosomes in the cardiovascular science were published in 428 academic journals. The journal of *Circulation Research* (46, 4.43%) had the highest number of outputs, followed by *International Journal of Molecular Sciences* (34, 3.27%), *Circulation* (28, 2.69%), *Frontiers in Physiology* (25, 2.41%), and *Scientific Reports* (22, 2.12%). Among the top 15 journals, *Circulation* has the highest impact factor (IF: 29.690), followed by *Circulation Research* with an IF of 17.367. The analysis of the distribution of the source of published articles is helpful to identify core journals.

Co-citation analysis is designed to measure the degree of relationship between articles. The impact of a journal depends on its co-citation frequency, which reflects the influence of a journal in a specific research field. Among 4,380 co-cited journals, 11 journals were cited over 1,000 times. As is shown in Table 2, *Circulation Research* (3,597) was the most frequently cited journal, followed by *Circulation* (2,299) and *Plos one* (2,221). Among the top 15 journals, *Nature* had the highest IF (49.962), followed by *Cell* with an IF of 41.582. According to the journal citation reports partition in 2020, almost all the co-cited journals were distributed in the Q1 region among the top 15 journals, except for *The Journal of Molecular and Cellular Cardiology*.

**Table 2:** Top 10 journals and co-cited journals related to exosomes in CVDs.

**No**.	**Journal**	**Count (%)**	**IF (2020)**	**JCR**	**Co-cited journal**	**Citation**	**IF (2020)**	**JCR**
1	Circulation research	46 (4.43%)	17.367	Q1	Circulation research	3,597	17.367	Q1
2	International journal of molecular sciences	34 (3.27%)	5.923	Q2	Circulation	2,299	29.690	Q1
3	Circulation	28 (2.69%)	29.690	Q1	Plos one	2,221	3.240	Q1
4	Frontiers in physiology	25 (2.41%)	4.566	Q2	Proceedings of the national academy of sciences of the United Stated of America	1,387	9.580	Q1
5	Scientific reports	22 (2.12%)	4.379	Q1	Journal of extracellular vesicles	1,338	25.841	Q1
6	Plos one	21 (2.02%)	3.240	Q1	Cardiovascular research	1,293	10.787	Q1
7	Journal of cellular and molecular medicine	21 (2.02%)	5.310	Q1/Q2	Nature	1,107	49.962	Q1
8	Theranostics	19 (1.83%)	11.556	Q1	Journal of biological chemistry	1,106	5.157	Q1
9	Stem cells research and therapy	18 (1.73%)	6.832	Q1/Q2	Scientific reports	1,069	4.379	Q1
10	Cardiovascular research	17 (1.64%)	10.787	Q1	Journal of the American college of cardiology	1,029	24.094	Q1
11	Journal of cardiovascular translational research	17 (1.64%)	4.132	Q2	Journal of clinical investigation	1,000	14.808	Q1
12	American journal of physiology-heart and circulatory physiology	17 (1.64%)	4.733	Q2	Blood	981	22.113	Q1
13	Frontiers in cell and developmental biology	13 (1.25%)	6.684	Q1/Q2	The journal of molecular and cellular cardiology	941	5.000	Q2
14	Frontiers in cardiovascular medicine	13 (1.25%)	6.050	Q2	Cell	874	41.582	Q1
15	Journal of molecular and cellular cardiology	12 (1.15%)	5.000	Q1/Q2	European heart journal	859	29.983	Q1

*IF, Impact Factor; JCR, Journal Citation Reports*.

**Figure 5:**
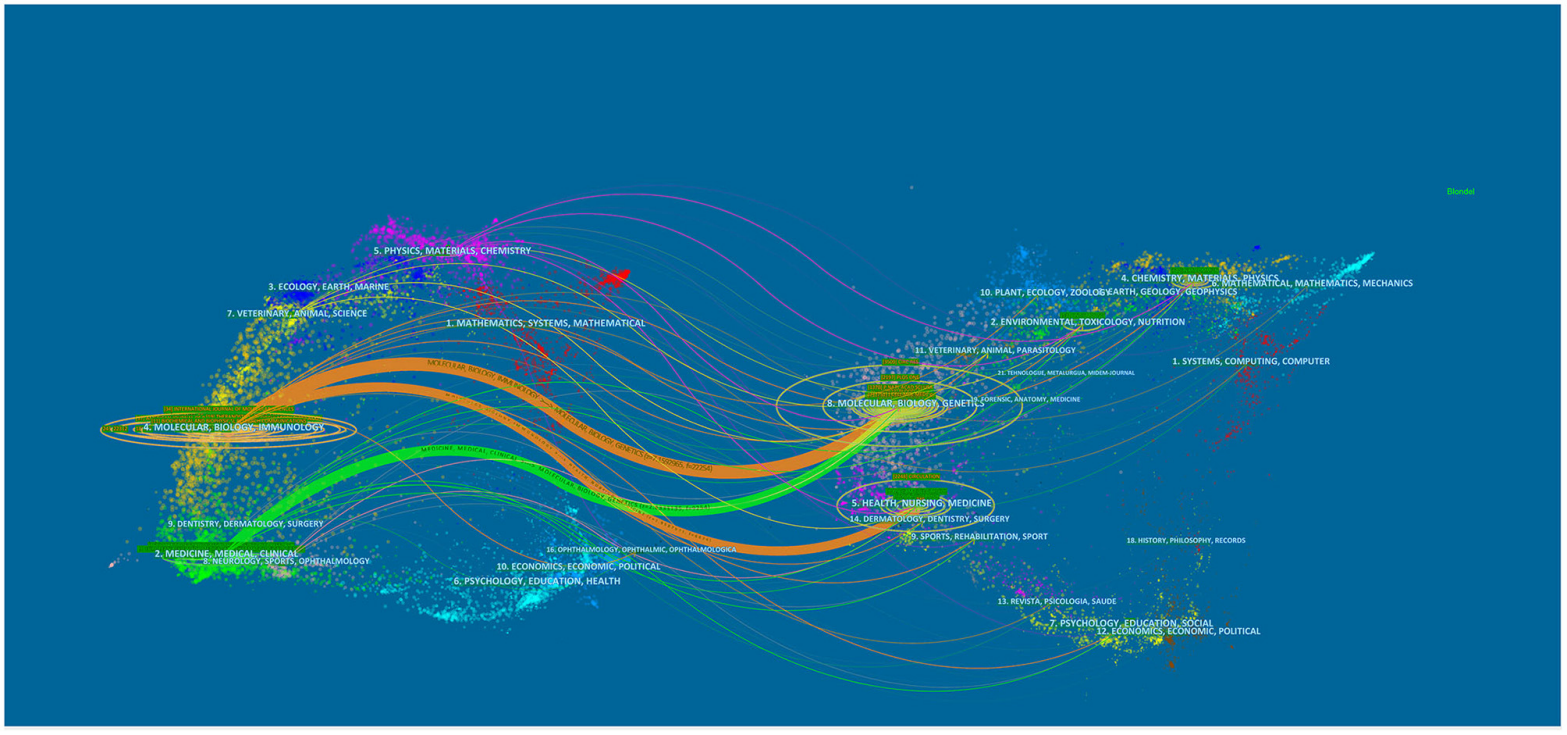
The dual-map overlay of journals on exosomes in CVDs.

The dual-map overlay of journals shows the distribution of relationships between journals, citing journals on the left and cited journals on the right. The colored paths between them indicate the cited relationships. As is shown in Figure 5, there are three main citation paths, including two orange paths and one green path. The orange path indicates that studies published in Molecular/Biology/Genetics journals and Health/Nursing/Medicine journals are cited for studies in Molecular/Biology/Immunology journals. The green path means that the studies published in Molecular/Biology/Immunology journals are generally cited by Medicine/Medical/Clinical journals.

### Authors and Co-cited Authors

**Figure 6:**
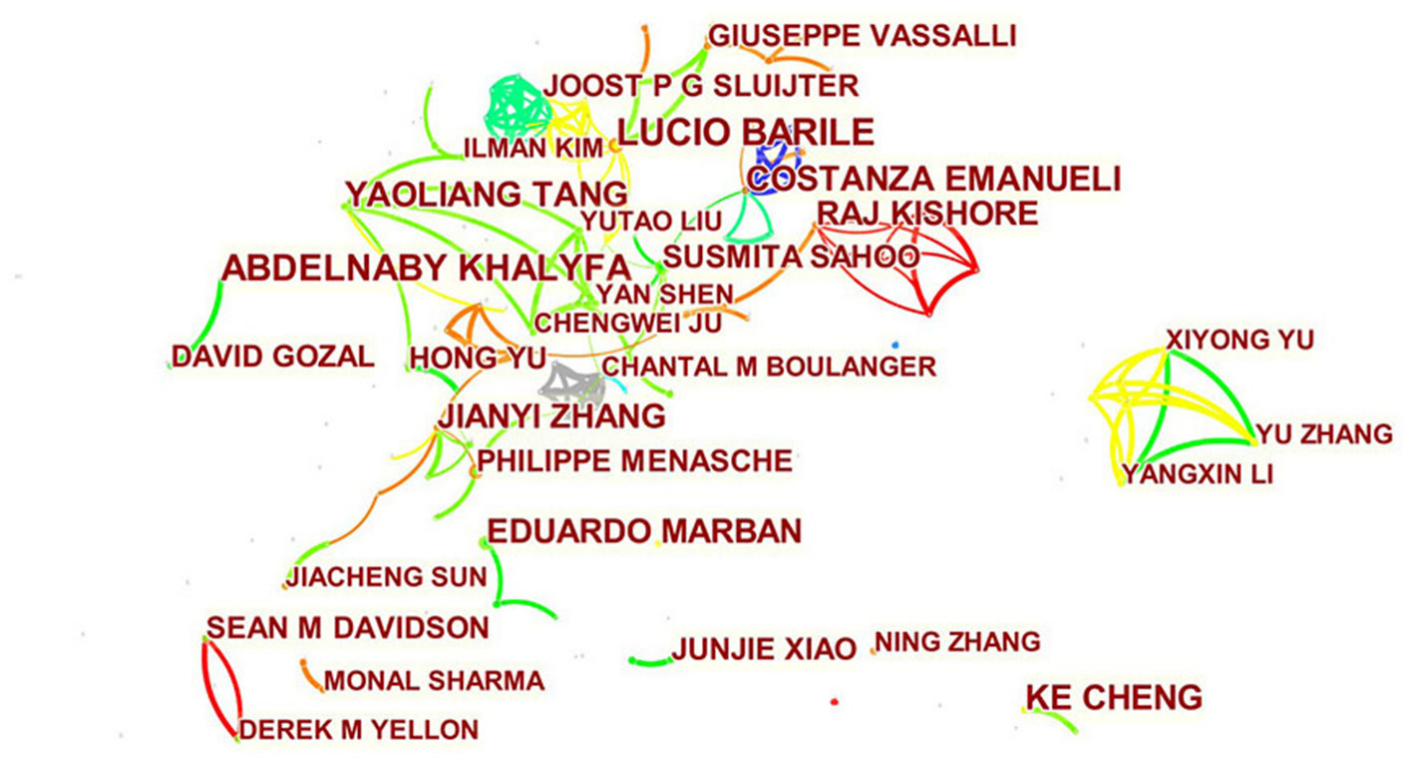
CiteSpace visualization map of authors involved in exosomes in CVDs.

**Figure 7:**
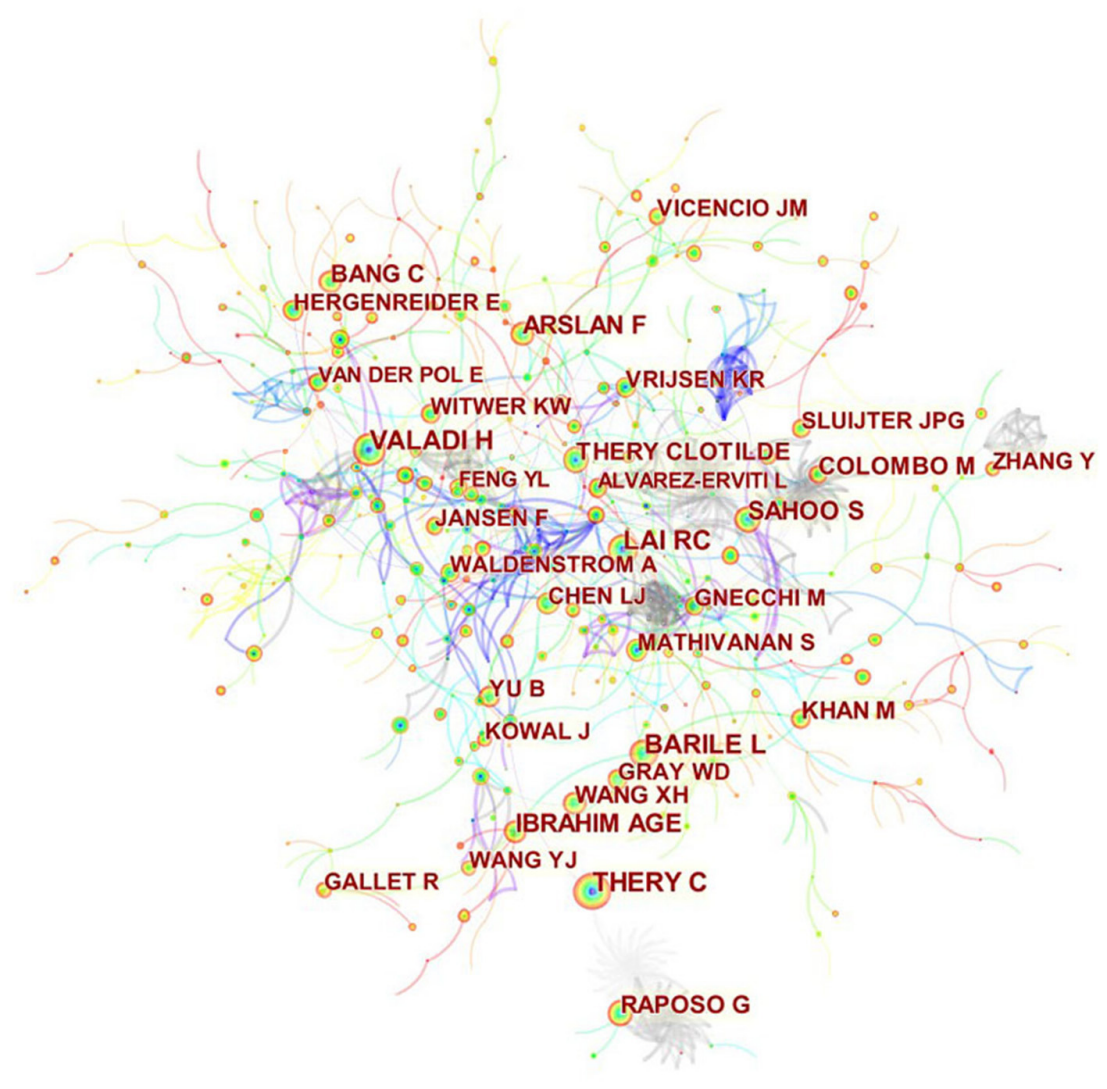
CiteSpace visualization map of co-cited authors involved in exosomes in CVDs.

A total of 473 authors published articles on exosomes in cardiovascular medicine (Table 3). Lucio Barile from Cardiocentro Ticino Laboratory for Cardiovascular Theranostics had the highest number of published papers (12, 1.15%), followed by Abdelnaby Khalyfa (10, 0.96%), Ke Cheng (9, 0.87%), Yaoliang Tang (9, 0.87%), and Costanza Emanueli (9, 0.87%). It is worth noting that the centrality of the authors is relatively low (≤0.03), suggesting that the influence of the authors on exosomes in cardiovascular science needs to be improved. Each node represents an author, with larger nodes representing more published articles. Thicker lines represnt closer cooperation between authors. Different colors refer to clusters of close cooperation. As shown in Figure 6, there was a communication and cooperation network among authors in this research area. Two or more authors that are cited simultaneously are called co-cited authors (Figure 7). Of the 803 co-cited authors, only four had a citation frequency of more than 200 times.

**Table 3:** Top 10 authors and co-cited authors related to exosomes in CVDs.

**No**.	**Author**	**Count (%)**	**Centrality**	**Co-Cited Author**	**Citation**	**Centrality**
1	Lucio Barile	12 (1.15%)	0.02	Thery C	268	0.09
2	Abdelnaby Khalyfa	10 (0.96%)	0.00	Lai RC	244	0.05
3	Ke Cheng	9 (0.87%)	0.00	Valadi H	238	0.05
4	Yaoliang Tang	9 (0.87%)	0.01	Barile L	237	0.03
5	Costanza Emanueli	9 (0.87%)	0.01	Sahoo S	179	0.03
6	Jianyi Zhang	8 (0.77%)	0.03	Ibrahim AGE	175	0.03
7	Raj Kishore	8 (0.77%)	0.01	Arslan F	161	0.01
8	Eduardo Marban	8 (0.77%)	0.00	Raposo G	159	0.06
9	Susmita Sahoo	7 (0.67%)	0.01	Bang C	158	0.02
10	Giuseppe Vassalli	6 (0.58%)	0.00	Wang XH	156	0.01

### Co-cited References and References Burst

Co-citation analysis indicated that two references appeared in the reference list of a third citation article, and then the two references formed a co-citation relationship. We listed the 12 most frequently cited references related to research on exosomes in cardiovascular medicine. Among the 834 cited references, 12 references were cited more than 100 times, and the references listed in the top three were all cited more than 150 times (Table 4). The most frequently cited reference topic was *Exosomes as Critical Agents of Cardiac Regeneration Triggered by Cell Therapy*, the basic experiment describing exosomes as critical factors for cardiac regeneration and cardiac protection in the paracrine pathway of stem cells.

**Table 4:** Top 12 co-cited references related to exosomes in CVDs.

**No**.	**Reference**	**Citation**	**Year**	**Centrality**
1	Exosomes as critical agents of cardiac regeneration triggered by cell therapy	175	2014	0.03
2	Mesenchymal stem cell-derived exosomes increase ATP levels, decrease oxidative stress and activate PI3K/Akt pathway to enhance myocardial viability and prevent adverse remodeling after myocardial ischemia/reperfusion injury	159	2013	0.03
3	Extracellular vesicles from human cardiac progenitor cells inhibit cardiomyocyte apoptosis and improve cardiac function after myocardial infarction	158	2014	0.01
4	Cardiac fibroblast–derived microRNA passenger strand-enriched exosomes mediate cardiomyocyte hypertrophy	146	2014	0.02
5	Embryonic stem cell-derived exosomes promote endogenous repair mechanisms and enhance cardiac function following myocardial infarction	141	2015	0.02
6	Exosome secreted by MSC reduces myocardial ischemia/reperfusion injury	123	2010	0.08
7	Extracellular vesicles: Exosomes, microvesicles, and friends	118	2013	0.02
8	Cardiac progenitor-derived exosomes protect ischemic myocardium from acute ischemia/reperfusion injury	118	2013	0.04
9	Identification of therapeutic covariant microRNA clusters in hypoxia treated cardiac progenitor cell exosomes using systems biology	114	2015	0.03
10	Exosomes secreted by cardiosphere-derived cells reduce scarring, attenuate adverse remodeling, and improve function in acute and chronic porcine myocardial infarction	106	2017	0.02
11	Plasma exosomes protect the myocardium from ischemia-reperfusion injury	102	2015	0.01
12	Biogenesis, secretion, and intercellular interactions of exosomes and other extracellular vesicles	100	2014	0.00

**Figure 8:**
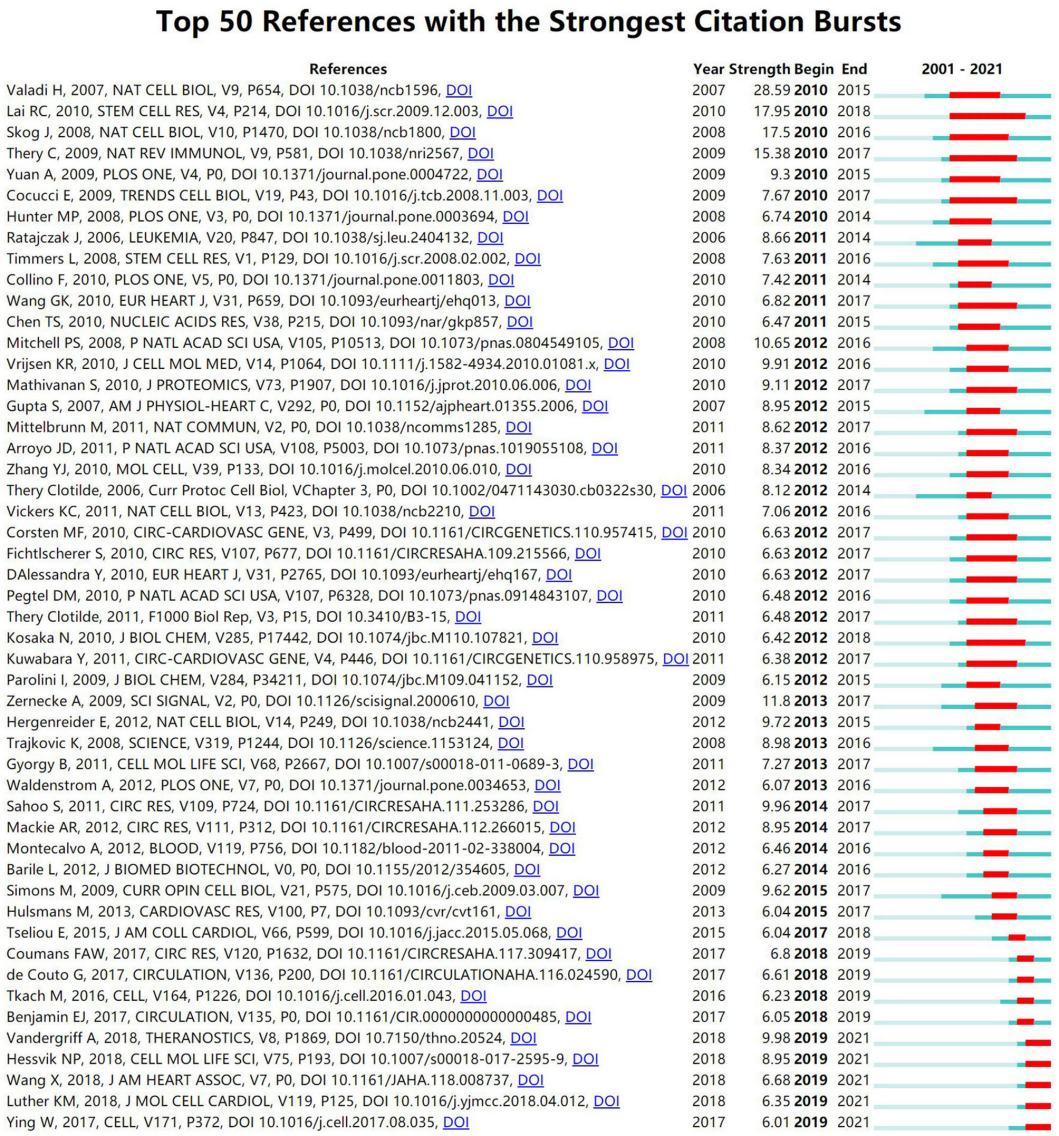
CiteSpace visualization map of top 50 references with the strongest citation bursts involved in exosomes in CVDs.

Figure 8 shows the top 50 references with the most robust citation bursts. It can be seen that the first reference with citation bursts was in 2010. Almost all references on the exosomes in cardiovascular science focused on the reliable citation power in the past 10 years, suggesting that this research may continue to expand in the future.

### The Analysis of Hotspots and the Frontiers

Keywords summarize research topics. Through the analysis of keywords, we can understand the research hotspots in specific fields. Table 5 shows the high-frequency keywords. Among these keywords, MI and microRNA occurred over 200 times, suggesting that exosomes in CVDs may hold substantial research potential.

**Table 5:** Top 20 keywords related to exosomes in CVDs.

**No**.	**Keywords**	**Count**	**Centrality**	**No**.	**Keywords**	**Count**	**Centrality**
1	Exosome	721	0.13	11	Stem cell	128	0.01
2	Extracellular vesicle	332	0.01	12	Angiogenesis	118	0.03
3	Myocardial infarction	266	0.08	13	Heart failure	115	0.02
4	MicroRNA	222	0.03	14	Therapy	102	0.03
5	Microvesicle	184	0.04	15	Mechanism	101	0.03
6	Mesenchymal stem cell	167	0.05	16	Endothelial cell	100	0.03
7	Heart	147	0.03	17	Cell	91	0.03
8	Cardiovascular disease	147	0.10	18	Inflammation	86	0.02
9	Biomarker	139	0.02	19	*In vitro*	75	0.04
10	Expression	136	0.09	20	Regeneration	71	0.04

**Figure 9:**
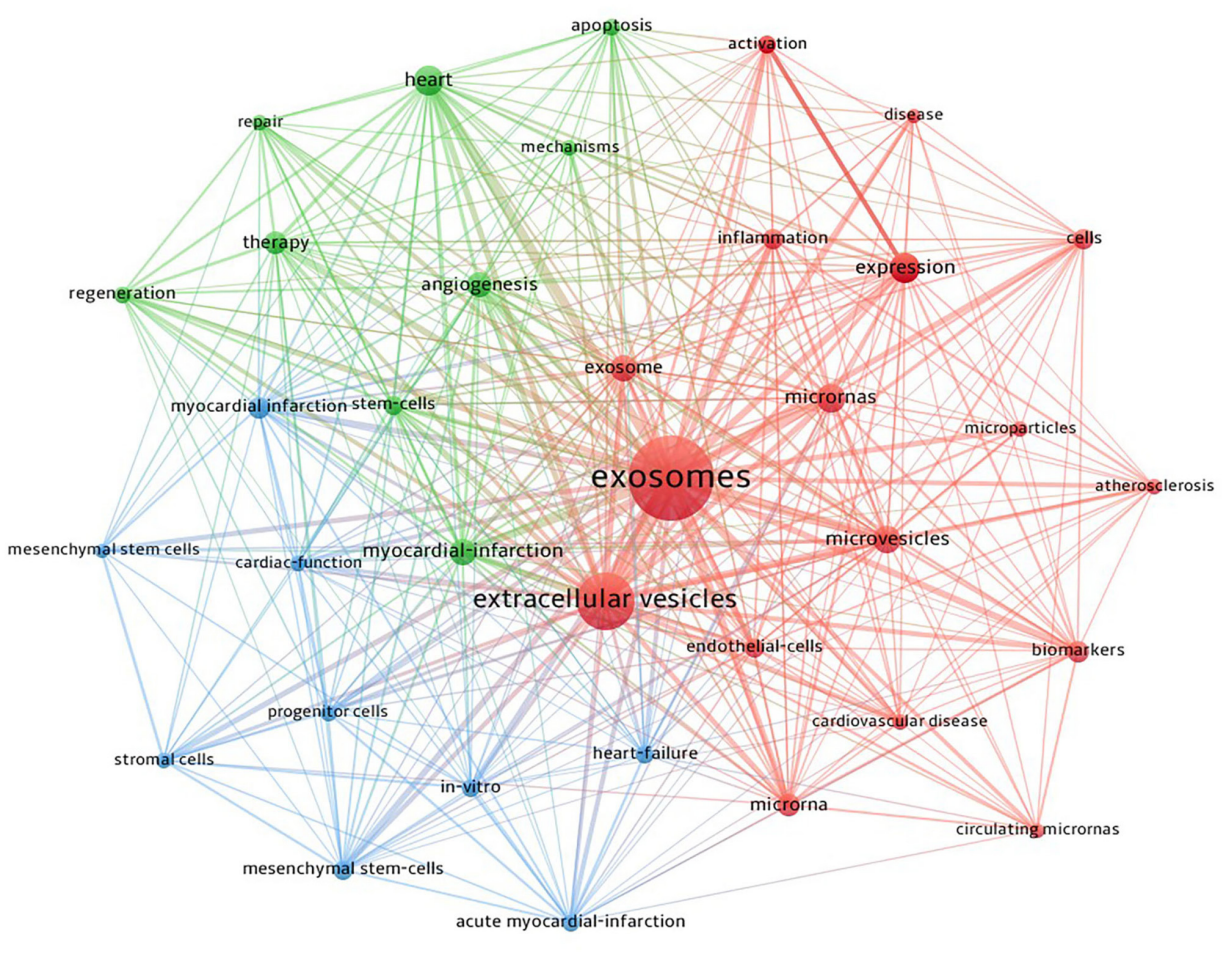
CiteSpace visualization map of keywords clustering analysis on exosomes in CVDs.

We used VOSviewer software to cluster the keywords. The circle and label form an element, the color of which identifies the cluster to which it belongs. Figure 9 displays the clusters of red, blue, and green, indicating three research directions. Green clusters are composed of exosome, CVD, atherosclerosis, biomarkers, and inflammation. The keywords of the red cluster are MI, MSCs, progenitor cells, therapy, repair, regeneration, angiogenesis, cardiac function, and *in vitro*. The keywords of the blue cluster are heart failure, microRNA, cells, expression, activation, apoptosis, and mechanism.

**Figure 10:**
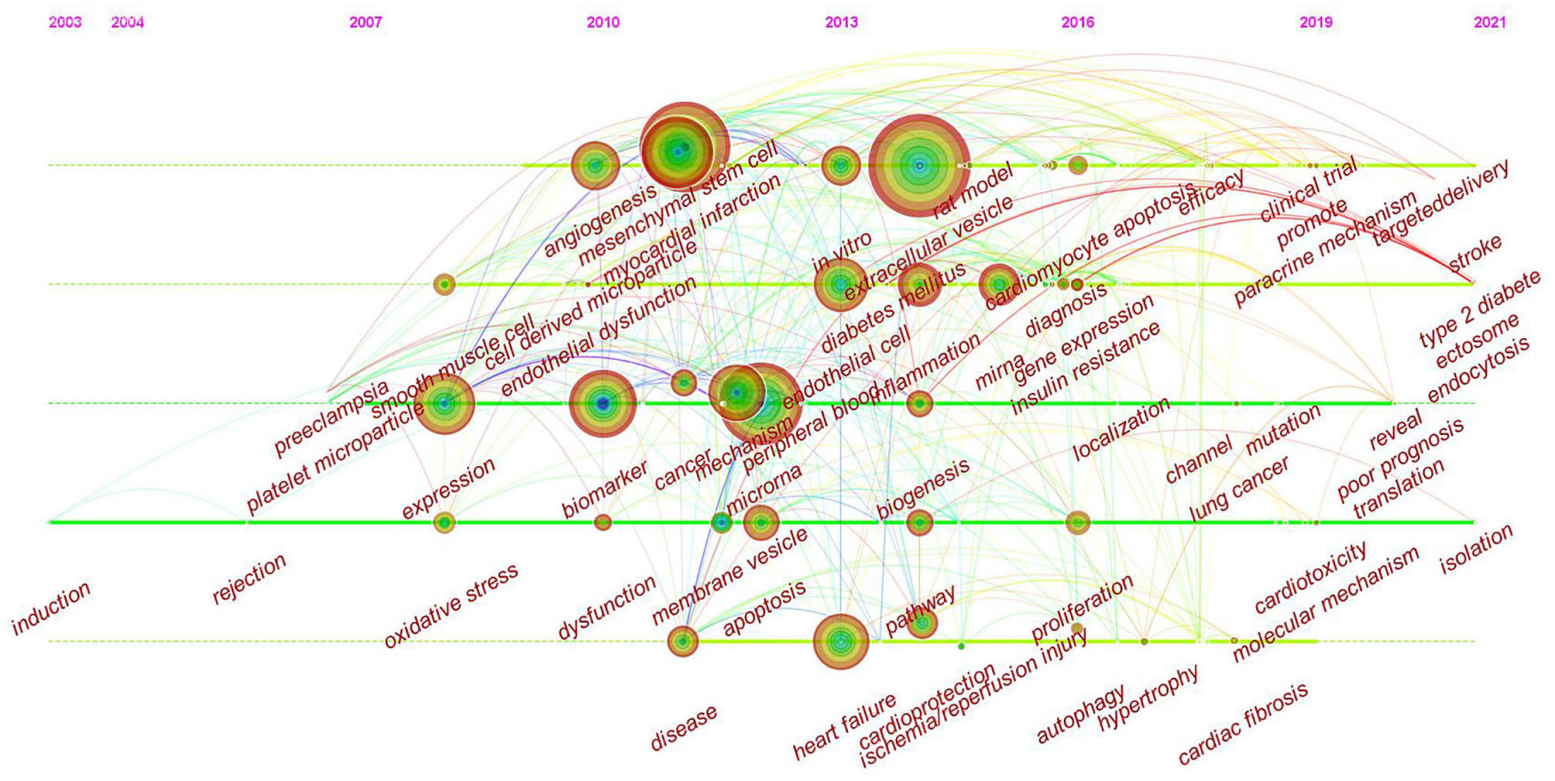
CiteSpace visualization map of timeline viewer related to exosomes in CVDs.

The timeline view is designed based on the interaction and mutation relationship between keywords in a particular field, and it helps to explore the evolutionary track and stage characteristics of the field. Figure 10 shows a timeline chart of exosomes in CVDs drawn based on CiteSpace software; it visually reflects the phased hotspots and developmental path of exosomes in CVD research from the time dimension. From 2003 to 2010, research on exosomes in cardiovascular science had not yet garnered attention. The research during this period was focused on the cell and extracellular vesicle levels. The main keywords were induction, platelet microparticle, expression, oxidative stress, and smooth muscle cell. From 2010 to 2019, research on exosomes in cardiovascular science increased, and related mechanism research accelerated. The main keywords were biomarker, MI, MSCs, angiogenesis, cancer, diabetes mellitus, peripheral blood, apoptosis, pathway, miRNA, diagnosis, inflammation, heart failure, hypertrophy, cardioprotection, ischemia/reperfusion injury, cardiac fibrosis, and gene expression. In the past 2 years, scholars began to explore the potential and value of exosomes in clinical research. The main keywords were a clinical trial, targeted delivery, stroke, poor prognosis, translation, isolation, and cardiotoxicity.

## Discussion

### General Information

The trend of annual publications published from 2001 to 2006 demonstrated that the studies during this period were lacking, suggesting that research on exosomes in CVDs was not in-depth and lacked a research basis between exosomes and disease. From 2007 to 2010, small numbers of articles began to appear; however the research during this period was embryonic. From 2010 to 2016, the number of articles began to increase, and a growing number of investigators began to pay attention to the role of exosomes in cardiovascular science (40–45). In the past 5 years, the number of published articles grew rapidly, suggesting that the research of exosomes is becoming more mature and its research in cardiovascular science is likely to become a hot topic and research direction in the future.

Using visual analysis of the distribution of countries and institutions, we can see that the US and China are the leading countries where research on exosomes of CVDs is occurring. Among the top 10 study institutions, nearly all research institutions were in these two countries, including Shanghai Jiao Tong University, Nanjing Med University, Temple University, and the University of Alabama Birmingham. As shown in Figures 3, 4, although countries possess their cooperation networks, the breadth, and intensity of cooperation were not ideal. For example, the US and China do not cooperate or communicate. From the perspective of research institutions, most cooperating institutions are limited to internal connections, and there is substantially less transnational cooperation and exchange of findings. This situation hinders the development of this research field. Therefore, it is strongly suggested that the US and China and research institutions from other countries should remove academic barriers, cooperate, and communicate to promote the research and development of exosomes in cardiovascular science.

Several studies of exosomes were published in influential cardiovascular journals such as *Circulation Research*, and *Circulation;* however, exosomes also have potential value based on other journals. For example, in addition to research on CVDs, exosomes in cardiovascular science also involve molecular science (46), pharmacology (47), therapeutics (48), stem cells (49), and heart transplantation (50). Regarding co-cited academic journals, we can see that the majority of studies are from high-impact journals. These journals have a significant influence on the international community and affect the research direction of their respective academic fields. In Figure 5, the dual-map overlay of journals indicates the subject distribution of academic journals. Considering the two main pathways in the diagram, research on exosomes in CVDs is limited to basic research and begins to convert primary research results into clinical research.

From the perspectives of author contributions and co-cited authors, Lucio Barile, a Swiss scholar, made the most contributions with 12 published articles, followed by Abdelnaby Khalyfa from the University of Chicago with 10 articles. It should be noted that Barile had the most significant number of published articles and ranked fourth among the co-cited authors. Barile's team studies exosomes, stem cells (51) and cardiac protection (52). In a frequently cited animal study, this group showed that exosomes derived from cardiac-resident progenitor cells might have a better cardioprotective effect than exosomes originated in marrow-derived MSCs, and the exosomes can play a role by binding related active proteins on its surface to ligands, which will provide a basis for future research on the cardioprotective effects of stem cell-derived exosomes (53). In 2020, Barile showed that during heart transplantation, plasma-derived circulating exosomes combined with endocardial, myocardial biopsy could be used as biomarkers to diagnose allograft rejection according to the differential protein spectrum on the membrane surface (13). These articles suggest that exosomes are likely to become physiological or pathological diagnostic markers in clinical practice in addition to their cardioprotective therapeutic effect. Among co-cited authors, Thery (268) was the most frequently cited, followed by Lai (244), Valadi (238), and Barile (237). One point that is often overlooked is that, although the number of articles and citations of contributing authors and co-cited authors is significant, their influence in this research field remains insufficient. Therefore, in the future, we should pay attention to the number of articles and improve the impact of the results.

There were more than 100 top 12 co-cited references. The top three co-cited references (15, 54, 55) focused on the effects of stem cell-derived exosomes on MI. These authors found cardioprotective effects of exosomes on MI, including reducing cardiomyocyte apoptosis, promoting revascularization, and reducing the size of MI. The most explosive reference was published in *Nature Cell Biology* by Valadi's group (54). The authors showed that exosomes could be used as signal carriers to mediate cell communication by transporting bioactive substances such as RNA and proteins. In recent years, the references with the highest citation frequency focused on exosome engineering (56), targeted therapy (57), and regenerative medicine (58). It can be seen that some breakthroughs have been made in the research of exosomes in cardiovascular science using advanced technology.

### The Hotspots and Frontiers

Keywords summarize research topics and core content. Based on keyword co-occurrence analysis, the distribution and development of various research hotspots in a particular field can be understood. In addition to exosomes and CVDs, the keywords that frequently appear in Table 5 are MI, microRNA, MSC, biomarker, and expression. Clustering was performed based on keyword co-occurrence analysis to obtain a clustering map of exosome keywords in CVDs (Figure 9), and finally, the clusters of three colors were formed.

According to clustering analysis of keywords and the timeline viewer (Figure 10), research on exosomes in the cardiovascular science mainly focused on the following aspects:

#### Role of Exosomes Derived From Stem Cells After MI

As regenerative medicine is approaching clinical applicability, to identify cardioprotection strategies to regenerate lost myocardium and restore cardiac function, cellular therapy for CVDs has become an active area of research (59). Stem cells potentially proliferate and differentiate, with self-renewal and replication, producing highly differentiated functional cells (60, 61). However, some obstacles to therapy include immunological incompatibility, complex operation, high cost, cancers risks, and the potential to form ectopic tissue (62). Several studies reported that the transfusion of stem cells could cause arrhythmias, pulmonary embolism, and vascular obstruction to varying degrees, resulting in the death of animals or patients. Poor engraftment and biosafety of stem cells directly affect the clinical application, becoming a significant obstacle to its commercial development (63, 64). Recently, evidence has suggested that stem cells exert their therapeutic effects in a paracrine manner, primarily through exosomes to enhance cell survival, differentiation, and adaptive immune responses. Exosomes without self-replicating capabilities have a lower risk of ectopic differentiation, tumor formation, genetic instability, and immune system rejection.

MI refers to the local myocardial ischemia and anoxic necrosis caused by coronary artery occlusion and blood flow interruption. Restoration of blood supply is the hallmark of the treatment of ischemic diseases, and a critical treatment is the promotion of angiogenesis. Several lines of evidence demonstrated that stem cells such as cardiosphere-derived cells, embryonic stem cells, MSCs, induced pluripotent stem cells (iPSCs), stem cells derived from adipose tissue, and derived exosomes could promote angiogenesis. Stem cell-derived exosomes contain pro-angiogenic factors or angiogenic-related regulatory factors to promote angiogenesis. Investigators can manipulate exosomes such as cargo carriers to transport biological molecules to target cells to promote angiogenesis by exosomes engineering or treating parent cells differently (65). Gao et al. found that exosomes derived from human iPSCs promote endothelial cell tube formation and microvessel sprouting from mouse aortic rings and protect human iPSCs-cardiomyocytes by reducing apoptosis, maintaining intracellular calcium homeostasis increasing adenosine 5′-triphosphate (66). Another animal study showed that exosomes derived from embryonic stem cells enhanced cardiac functional recovery after MI and promoted angiogenesis, improving myocardial cell survival and reducing myocardial fibrosis. Notably, 8 weeks after *in vivo* transplantation, embryonic stem cell-derived exosomes enhanced the survival and proliferation of cardiac progenitor cells to form new cardiomyocytes in ischemic areas (67). These findings suggest that stem cell-derived exosomes promote angiogenesis, exert anti-inflammatory and cardioprotective effects, reduce apoptosis, and inhibit fibrosis.

Although exosomes provide therapeutic opportunities for ischemic diseases, especially MI, several limitations restrict their clinical application. First, the production, isolation, storage of exosomes, and culture conditions of their parent cells lack optimal protocols and remains a lack of reliable potency assays to evaluate the efficacy of exosomes therapy. Researchers have used several methods to isolate exosomes; the most common are ultracentrifugation-based techniques and ultrafiltration; however, it appears that the isolation method affects experimental results, at least to some extent (68). Second, researchers need to pay attention to the health and physiological status of the parent cells that secrete exosomes. Rezaie et al. reported MSCs and their exosomes kinetic are affected by aging and other aged exosomes. Exosomes from aged MSCs lose their regeneration potential, accelerating biological development and negatively impacting the function of recipient cells (69). Choosing an appropriate pathway for exosomes to access target sites is another issue that needs to be addressed. Biomaterials such as hydrogels have been developed to deliver large doses of exosomes to target tissues to induce angiogenesis to overcome the disadvantages of faster clearance by the intravenous injection route, considered the most widely used. Optimizing the administration method to obtain high efficacy and specificity to treat specific diseases is critical to the clinical application of exosomes (70). Finally, the uptake ability of target cells may also affect the treatment effects of exosomes.

Regarding these, exosomes originating from stem cells have attracted considerable attention in tissue engineering and regeneration. Nevertheless, there is a paucity of large-scale clinical data to verify this. Further research is needed to overcome these limitations in the future (65).

#### Function and Mechanism of Exosomes in CVDs

Nearly all cell types can spontaneously create or produce exosomes under certain stimuli, and the generation of exosomes involves double invagination. The first invagination of exosomes occurs *via* inward budding of the plasma membrane to form a cup-shaped intracellular endosomal compartment that contains cell-surface proteins, fluid, and extracellular constituents such as proteins, lipids, and metabolites (71). Further invagination of the intracellular endosomes forms multivesicular bodies (MVBs) that contain specific intraluminal vesicles of various sizes and specific content. MVBs are either degraded by fusing with lysosomes or autophagosomes or fusing with the plasma membrane to release their contents into the extracellular microenvironment as exosomes (72, 73). These findings suggest that exosomes promote cell-to-cell communication by carrying cargos of origin to transfer signals from one cell to another. The heart is a complex of different cells, including cardiomyocytes, cardiac progenitor cells, endothelial cells, fibroblasts, vascular smooth muscle cells, and immune cells. Intercellular communication and crosstalk maintain the homeostasis and function of the heart and accelerate the pathological process of various types of CVDs (74, 75). Zheng et al. found that vascular smooth muscle cell-derived exosomes mediate the transfer of Krüppel-like factor 5-induced miR-155 from smooth muscle cells to endothelial cells, damaging the tight junction of endothelial cells and the integrity of barriers leading to increased endothelial cell permeability and accelerating atherosclerosis (76). Macrophage-derived exosome miR-21-3p, which has been treated with nicotine, the principal component of cigarette smoke, may accelerate the development of atherosclerosis by increasing the migration and proliferation of vascular smooth muscle cells through its target protein (77). Exosomes mediate the occurrence and development of CVDs *via* upstream or downstream signaling pathways *in vivo*, and the relevant mechanisms are being elucidated (78–80).

Exosomes are used to develop and deliver drugs for therapeutic purposes. Many synthetic drug delivery systems have been developed and introduced to the market over the past few decades; however, their application has been limited due to inefficiency, cytotoxicity, or immunogenicity. Natural drug carrier systems have developed rapidly, especially involving exosomes. Compared with synthetic drug delivery systems, exosomes can be distributed throughout the body and cross the blood-brain barrier by using natural intracellular transport capacity and biocompatibility. Akbari and Rezaie found that exosomes can be used as drug delivery systems for treating severe acute respiratory syndrome coronavirus 2 pneumonia. Exosomes have advantages over other nanocarriers in that they are phospholipid vesicles that are derived from cells. They are relatively safe with low immunogenicity and can pass through physiological barriers. In this respect, exosomes can be constructed by direct or indirect engineering. Direct engineering refers to loading therapeutic agents such as biomolecules or drugs directly into exosomes, and then these exosomes are delivered to target tissues. Indirect engineering refers to co-culture or genetic modification of parent cells and therapeutic agents to produce artificial/drug-loaded exosomes (62). Sun et al. demonstrated that encapsulating curcumin in exosomes enhanced anti-inflammatory activity (81). Tang and colleagues reported that inoculating chemotherapeutic drugs with tumor cells allows the drugs to be packaged as exosomes, and collecting these exosomes and using them in a mouse tumor model killed tumor cells with no significant side effects (82). Nevertheless, more efforts are needed to realize the transformation and application of exosomes. Clinically, it is necessary to overcome the difficulties in the production, isolation, and storage of exosomes and explore the mechanism of exosomes production, cargo classification, internalization, and transportation.

#### Application of Exosome as a Biomarker in CVDs

The gold standard for diagnosing coronary atherosclerosis and viral myocarditis remains invasive examination (83, 84), while MI and acute heart failure require specific and rapid diagnosis (85). Exosomes are secreted by endothelial cells, cardiac progenitor cells, cardiac fibroblasts, and cardiomyocytes, suggesting that exosomes are involved in CVDs. Exosomes secreted by damaged or diseased hearts can carry intracellular substances that may reflect cell origin and pathophysiological states as signatures or fingerprints of donor cells. In various clinical environments, substances such as RNA and proteins carried by exosomes may serve as prognostic and diagnostic markers of CVDs (86–89).

In the updated ExoCarta, several contents were identified in exosomes, including ~9,769 proteins, 3,408 mRNA, 2,838 miRNA, and 1,116 lipids. In CVDs, the content of exosomes can be altered according to the severity of the disease. A study showed that exosomes and circulating miRNAs were significantly increased after myocardial ischemia-reperfusion injury in pigs. Myocardial and muscle-specific miRNAs rapidly increased in plasma 2.5 h after ischemia, while the number of exosomes was increased as early as 1 h after ischemia. It was found that accumulated exosomes were enriched with miRNA-133b, miRNA-208b, and miRNA-499 (90). Cargo RNAs of exosomes contain various biotypes, including mRNA, miRNA, lncRNA, and circRNA. This diversity of RNAs, the heterogeneity of exosomes, and the overall low concentration of RNAs in exosomes complicate the characterization of RNA cargo of exosomes. More than 200 miRNAs exist in the heart, and cardiac-derived miRNAs participate in heart development and function regulation. In recent years, miRNAs have been shown to function as micromodulators in cellular communications and are involved in cell signaling and microenvironment remodeling. Exosomes containing miRNAs serve as biomarkers for the diagnosis and outcome prediction of CVDs (91–96). Studies showed that the cardiac specificity of miRNAs appears to be better than conventional diagnostic tests such as troponin because miRNAs can be detected more rapidly and are more sensitive and specific than myocardial troponin in the context of acute MI. Several studies showed that the miRNAs of heart-derived exosomes could be detected in urine, which opens the way for liquid biopsy in cardiovascular medicine (97–99).

Exosome as biomarkers have several advantages. They can travel in several body fluids and are involved in various pathophysiological processes throughout disease progression. They are also convenient to sample. More importantly, the lipid bilayer membrane of the exosome protects the miRNA from decomposition (100–103).

Research on exosomes in cardiovascular science is increasing exponentially, and the accumulated research contributes to the potential of exosomes as non-invasive clinical biomarkers. However, given further development of precision medicine, it is critical to consider individual differences' molecular characteristics, lifestyle, and environmental impact (99).

## Limitations

CiteSpace and VOSviewer cannot wholly replace system retrieval. The uneven quality of collected literature data can reduce the credibility of atlas drawing, and the differences of data update also cause the retrieval results to differ from the actual number of included articles. Therefore, more accurate literature analysis should be based on the knowledge map constructed by CiteSpace and VOSviewer combined with specific literature. Nevertheless, literature analysis based on visualization undoubtedly lays the foundation for scholars to quickly understand the research hotspots and development trends of exosomes in cardiovascular science.

## Conclusion

Exosomes possess essential research value and application prospects in cardiovascular science. Using CiteSpace and VOSviewer software for visual analysis, research on exosomes in CVDs demonstrated a substantial development trend. Increasing numbers of articles published in international core journals suggest a significant impact. The leading countries are the US and China; however, there is a need for enhanced cooperation and exchange between countries and institutions. All scholars should increase the number of articles and strengthen the influence of articles. In addition to focusing on basic research, we should focus on the transformation of results and the study of exosomes in patients with CVDs. At present, the research on exosomes in cardiovascular science focuses on ischemic heart disease, pathogenesis, regeneration, stem cells, targeted therapy, biomarkers, and cardiac protection, and these will serve as the focus of future research.

## Data Availability Statement

The original contributions presented in the study are included in the article/supplementary material, further inquiries can be directed to the corresponding author/s.

## Author Contributions

DM and SL conceived the study. BG, LS, and TW collected the data. DM, LZ, and ZZ re-examined the data. BG, QL, and YF analyzed the data. DM wrote the manuscript. ZG, SL, and HX reviewed and revised the manuscript. All authors contributed to the article and approved the submitted version.

## Conflict of Interest

The authors declare that the research was conducted in the absence of any commercial or financial relationships that could be construed as a potential conflict of interest.

## Publisher's Note

All claims expressed in this article are solely those of the authors and do not necessarily represent those of their affiliated organizations, or those of the publisher, the editors and the reviewers. Any product that may be evaluated in this article, or claim that may be made by its manufacturer, is not guaranteed or endorsed by the publisher.
